# P-522. Epidemiology of respiratory viral infections in pediatric acute leukemia patients

**DOI:** 10.1093/ofid/ofaf695.737

**Published:** 2026-01-11

**Authors:** Caitlin N Brammer, Trinity Lee, Hannah Bahakel, Hannah Kim, Grant C Paulsen, Hilary Miller-Handley, Lauren Pommert, Lara A Danziger-Isakov, William R Otto

**Affiliations:** Cincinnati Children's Hospital Medical Center, Cincinnati, OH; Cincinnati Children's, Northville, Michigan; Cincinnati Children's Hospital Medical Center, Cincinnati, OH; University of Cincinnati, Cincinnati, Ohio; Cincinnati Children's Hospital Medical Center, Cincinnati, OH; Cincinnati Children's Hospital Medical Center, Cincinnati, OH; Cincinnati Children's Hospital Medical Center, Cincinnati, OH; Cincinnati Children's Hospital, Cincinnati, OH; Cincinnati Children's Hospital Medical Center, Cincinnati, OH

## Abstract

**Background:**

Respiratory viral infections (RVIs) are a significant source of morbidity and mortality in children with acute lymphoblastic leukemia (ALL) and acute myeloid leukemia (AML). Due to currently limited data, this study sought to describe the incidence and outcomes of RVIs in children with acute leukemia.

**Table 1:**
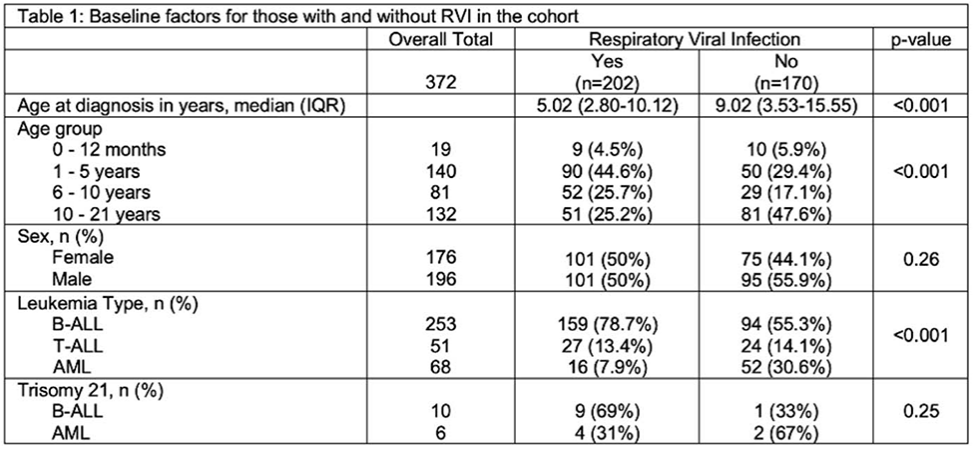
Baseline factors for those with and without RVI in the cohort

**Table 2:**
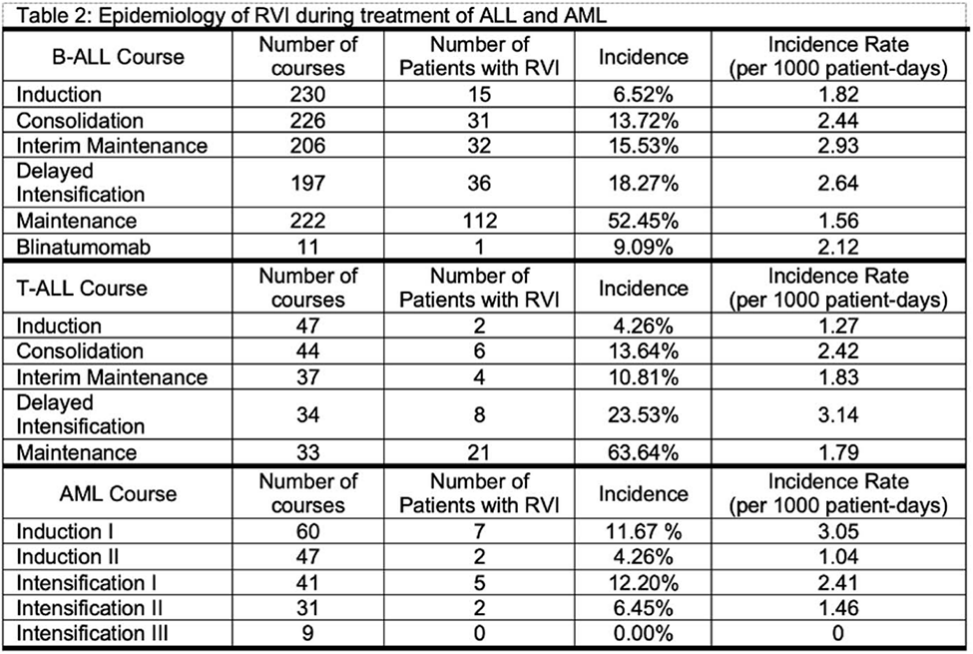
Epidemiology of RVI during treatment of ALL and AML

**Methods:**

This retrospective cohort study included patients with *de novo* leukemia treated at Cincinnati Children’s Hospital Medical Center from 2011-2022. Clinical and microbiological data were collected. The epidemiology and outcomes of RVIs were described. A mixed-effects regression model was developed to identify risk factors for lower respiratory tract infection (LRTI).

**Table 3.**
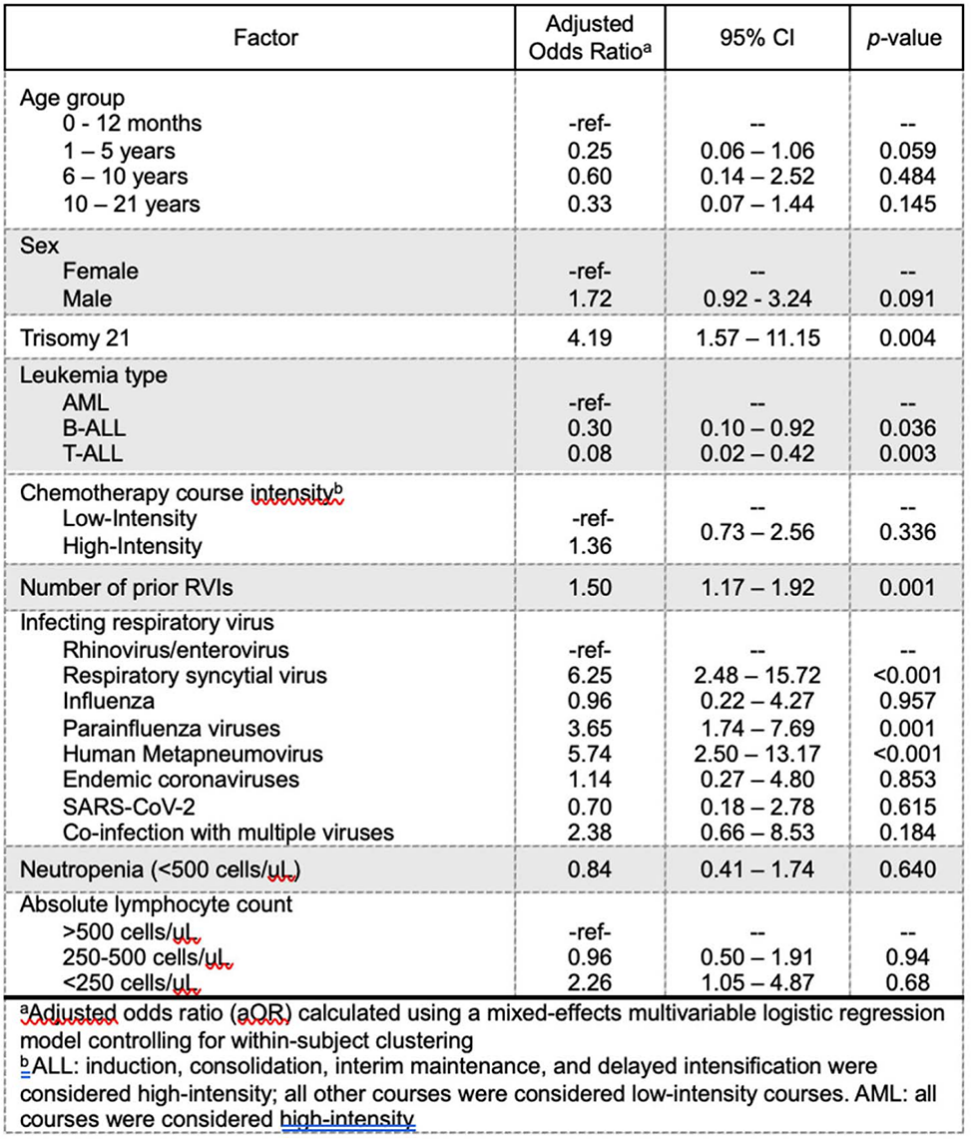
Factors associated with development of a lower respiratory tract infection

**Figure 1:**
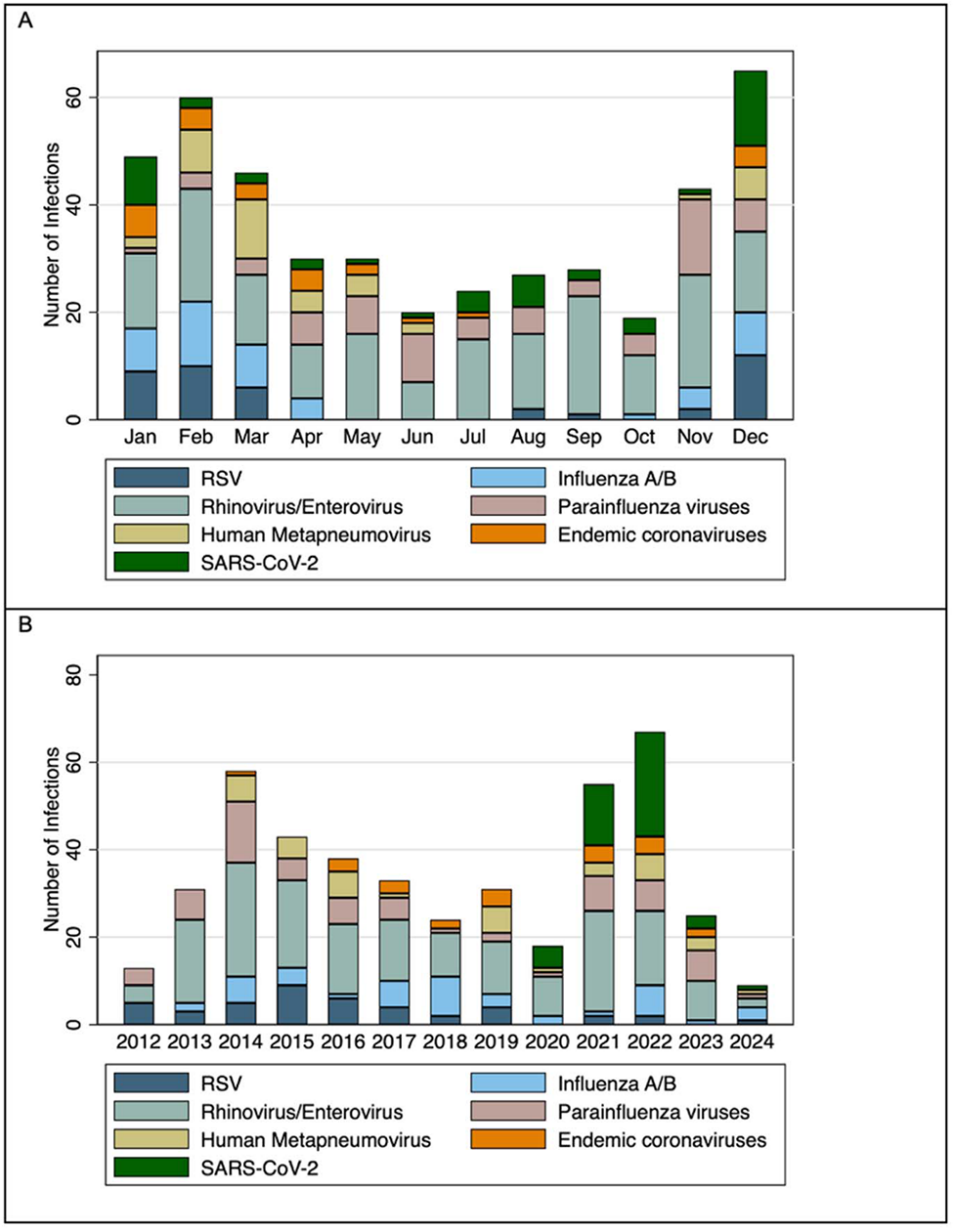
Occurrence of RVI by month and year. For episodes where co-infection with multiple viruses occurred, each virus was counted individually.

**Results:**

Among 372 patients, 202 (54.3%) developed 412 RVIs during treatment, including 159/253 (62.8%) of children with B-ALL, 27/51 (52.9%) for T-ALL, and 16/68 (23.5%) for AML. Children with RVIs were younger, more likely to have B-ALL, and more likely to have trisomy 21 (Table 1). Course-specific RVI incidence rates for ALL and AML are shown in Table 2. The majority of RVI episodes (268/412, 65.1%) occurred while hospitalized or required admission. A total of 40/412 (9.71%) episodes were LRTIs at presentation, and 21/412 (5.10%) progressed to LRTIs after diagnosis. Supplemental oxygen was required in 78/412 (18.93%) episodes, and 3/412 (0.73%) patients required mechanical ventilation. A total of 36/412 (8.7%) of episodes required intensive care, and two patients had long-term respiratory complications post-RVI. One death was attributable to RVI. The most frequent viruses were rhinovirus (37.38%), severe acute respiratory coronavirus 2 (SARS-CoV-2, 10.68%), and respiratory syncytial virus (8.98%). Respiratory viral infections were more common in winter and spring (Figure 1). The number of RVIs varied each year, with high numbers seen in 2021 and 2022 after the SARS-CoV-2 pandemic. Factors associated with development of a LRTI are shown in Table 4, including trisomy 21, number of prior RVIs, and infection with paramyxoviruses.

**Conclusion:**

In this pediatric leukemia cohort, RVIs were common, particularly in those with ALL and trisomy 21. Clinical outcomes were good, with few sequelae and low morbidity. This study provides important information regarding the epidemiology of RVI in this high-risk population.

**Disclosures:**

Grant C. Paulsen, MD, Moderna, Inc: Grant/Research Support|Pfizer: Grant/Research Support|Sanofi: Grant/Research Support Lara A. Danziger-Isakov, MD, MPH, Aicuris: Grant/Research Support|Ansun BioPharma: Grant/Research Support|Astellas: Advisor/Consultant|Astellas: Grant/Research Support|Merck: Advisor/Consultant|Merck: Grant/Research Support|Pfizer (Any division): Grant/Research Support|Takeda: Grant/Research Support

